# Milk Fat Globule Membranes for Mental Health across the Human Lifespan

**DOI:** 10.3390/foods13111631

**Published:** 2024-05-24

**Authors:** Rebecca Slykerman, Naomi Davies, Maher Fuad, James Dekker

**Affiliations:** 1 Department of Psychological Medicine, The University of Auckland, Auckland 1023, New Zealand; naomi.davies@auckland.ac.nz; 2 Fonterra Cooperative Group Limited, Palmerston North 4472, New Zealand; maher.fuad@fonterra.com (M.F.); james.dekker@fonterra.com (J.D.)

**Keywords:** milk fat globule membrane, mental health, gut–brain axis, depression, stress, anxiety, brain health

## Abstract

The milk fat globule membrane (MFGM) contains bioactive proteins, carbohydrates, and lipids. Polar lipids found in the MFGM play a critical role in maintaining cell membrane integrity and neuronal signalling capacity, thereby supporting brain health. This review summarises the literature on the MFGM and its phospholipid constituents for improvement of mental health across three key stages of the human lifespan, i.e., infancy, adulthood, and older age. MFGM supplementation may improve mental health by reducing neuroinflammation and supporting neurotransmitter synthesis through the gut–brain axis. Fortification of infant formula with MFGMs is designed to mimic the composition of breastmilk and optimise early gut and central nervous system development. Early behavioural and emotional development sets the stage for future mental health. In adults, promising results suggest that MFGMs can reduce the negative consequences of situational stress. Preclinical models of age-related cognitive decline suggest a role for the MFGM in supporting brain health in older age and reducing depressive symptoms. While there is preclinical and clinical evidence to support the use of MFGM supplementation for improved mental health, human studies with mental health as the primary target outcome are sparce. Further high-quality clinical trials examining the potential of the MFGM for psychological health improvement are important.

## 1. Introduction

### 1.1. Milk Fat Globule Membranes

**Figure 1:**
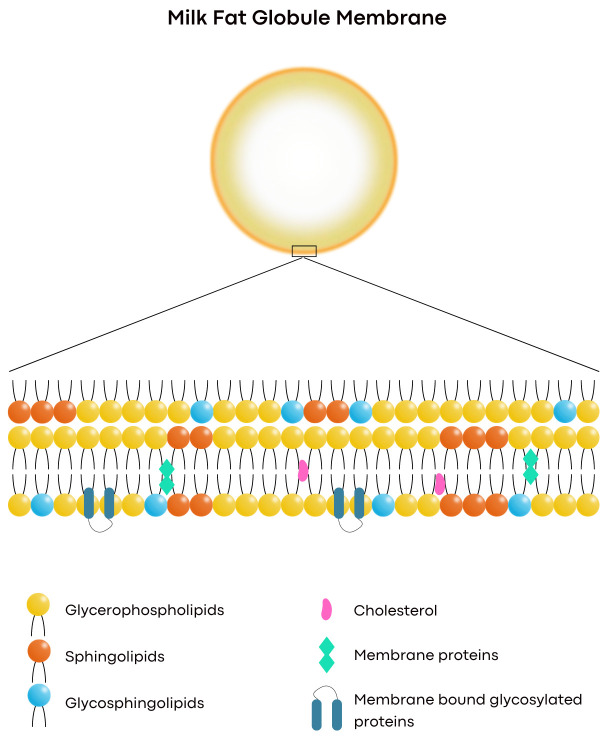
The milk fat globule membrane.

The milk fat globule membrane (MFGM) surrounds the fat globules present in mammalian milk. Synthesis of the membrane originates in the endoplasmic reticulum of the mammary cell, and the resulting membrane is organised in a three-layer structure comprised of lipids, proteins, and carbohydrates (Figure 1). Lipids within the MFGM are composed of polar and non-polar lipids. Non-polar lipids include cholesterol and glycerides. Phospholipids and sphingolipids are the main classes of polar lipids found in the MFGM and include phosphatidylcholine (PC), phosphatidylethanolamine (PE), phosphatidylinositol (PI), phosphatidylserine (PS), and sphingomyelin (SM) and gangliosides [1,2]. The MFGM is a rich source of polar lipids [3,4]. MFGMs in mammalian milk are compositionally similar but differ in their relative concentration of polar lipids. Human milk contains more SM than bovine milk or soy lecithin, while soy lecithin contains more PC and bovine milk contains more PS [5]. Factors including the stage in the lactation cycle and diet contribute to composition differences [4,6,7]. Bovine milk is the primary source of MFGM preparations in industry. MFGMs can be obtained from raw milk in the laboratory. Commercial MFGM preparations can be derived as a by-product from the manufacture of dairy products, including cheese, butter, buttermilk, and whey [8], where the exact composition of the product varies by the composition of the source ingredient [9] and processing method, such as homogenisation [10]. High-pressure homogenisation increases the SM content and decreases the amount of PC in the MFGM of milk. By contrast, standard homogenisation decreased the PE concentrations of the MFGM in buttermilk [9].

The potential health benefits of the MFGM extend from infant development to gut, brain, and immune function and improved physical endurance and cognition by providing bioactive nutrients [2,6,11,12,13]. Phospholipids are amphophilic lipids that are a building block of cellular membranes with a critical role in membrane health, integrity, and neuronal cell signalling capacity. Polar lipids are the most widely investigated component of the MFGM for enhanced health. However, the synergistic effects of MFGM components may exert the most significant benefit compared with isolated phospholipid constituents [14]. The brain is rich in lipids that play an essential role in maintaining membrane integrity, health, and the signalling capacity of cells [15,16]. Several components of the MFGM are bioactive, making the MFGM a potential functional dairy product with benefits for mental health [3].

### 1.2. Mental Health

Mental disorders present a significant modern-day issue. The increasing prevalence of mental health problems places a considerable burden on individuals, communities and broader society. By the age of 75 years, more than 50% of people will have experienced a mental health disorder, according to a global survey [17]. Mental health disorders increase the risk of non-communicable physical health illnesses and premature mortality [18]. The economic costs of mental health disorders extend far beyond the cost of mental and physical healthcare and include costs to individual productivity and contribution to the economy, with an estimated economic burden of USD 5 trillion [19,20]. Antidepressant medication is the standard treatment option for people diagnosed with Major Depressive Disorder, yet the efficacy of these medications remains under debate [21]. Safe, well-tolerated interventions are required to treat symptoms and lessen the long-term sequelae of mental illness. Nutritional interventions to prevent the worsening or development of psychiatric symptoms are equally important. Subclinical symptoms of depression, anxiety, or stress can interfere with daily functioning and wellbeing. The prevalence of subthreshold depression is estimated to be as high as 29% in adolescents [22], with estimates of 11% prevalence in US adults and associated increased risk of developing Major Depressive Disorder (MDD) [23]. Stress is an adaptive physiological and emotional response to challenging situations [24]. However, persistent or excessive stress is associated with an increased risk of mental health problems, most commonly with depression and anxiety. The connection between even mild stress and the risk of more significant mental health problems suggests that targeting stress is an essential point of intervention to prevent the development of mental health disorders [25].

In contrast to mental illness, mental health is a state of wellbeing in which an individual realizes their abilities, can cope with the everyday stresses of life, can work productively and can contribute to their community [26]. This definition of mental health expands the concept beyond the simple absence of a mental health disorder, creating potential for interventions to enhance mental wellbeing in healthy individuals in addition to preventing or treating mental disorders. Positive mental health and flourishing are associated with a reduced risk of all-cause mortality and development of later mental illness [27,28]. Promoting optimal mental health has positive benefits for individuals, communities, and society. 

This article reviews the relevant literature on milk fat globule membranes for mental health across three key stages of the human lifespan, childhood, adulthood, and older age. We summarise the current literature on the MFGM, and its phospholipid constituents for improved mental health spanning improvements in emotional development in infants and stress reduction in adults to the amelioration of symptoms specific to mood and anxiety disorders. The MFGM, like other dietary supplements, can target brain health encompassing both cognitive abilities and psychological health. Here, we focus on mental health as the term pertains to psychological health or psychiatric diagnoses rather than cognition. Disentangling cognition from mental health in the psychological sense can be complex particularly in older age where cognitive decline may be closely intertwined with mood and mental health. Where relevant, we discuss studies that include the assessment of cognitive functions that may also impact mental health. 

This article reviews recent pre-clinical and clinical literature about potential mechanisms by which the MFGM may improve mental health followed by use of the MFGM or phospholipids at key life stages. Finally, this article discusses research directions to further our knowledge of the application of MFGMs for improving mental health.

## 2. Mechanisms Underpinning MFGM for Improved Mental Health

Diet and nutrition are critical factors contributing to mental wellbeing [29]. As the growing field of nutritional psychiatry suggests, the food and supplements we ingest alter our physiology and contribute to mental health and disease. Consumption of dairy products is associated with a lower risk of depression [30,31,32]. Studies on the relationship between diet and adolescent depression suggest that intake of milk and dairy products reduces after childhood [33]; observational studies show that a high-quality diet is associated with a significant reduction in the risk of depression [34,35,36]. Intervention trials have further demonstrated that a Mediterranean style diet that includes nuts can reduce the risk of developing depression [37] and greater adherence to a Mediterranean style of eating may confer greater protection from the risk of developing depression as people age [38]. 

The gut microbiota can be conceptualised as a transdiagnostic modulator of the relationship between diet quality and psychological outcomes from infancy to old age [39,40]. Animal models suggest that up to 50% of the gut microbiota composition is due to diet [41]. Diet alteration can result in compositional changes within the human microbiota within days [42]. The physiological stress response is now understood to involve the gut–brain axis [43]. The immune, endocrine, and nervous system pathways that connect the central nervous system and gut microbiota in bidirectional communication are implicated in the psychological experience of stress and symptoms of low mood or anxiety [44]. People with different psychiatric disorders, including depression, anxiety, bipolar disorder and schizophrenia, have altered gut microbial composition with decreases in the abundance of anti-inflammatory butyrate-producing bacteria and increases in inflammatory markers present [45,46]. 

Neuroinflammation is an immune response mediated by microglia in the central nervous system (CNS). Persistent neuroinflammation has been implicated in mental disorders, including Major Depression [47], and can be altered by diet [48,49,50,51]. A cafeteria-style diet high in sugar and fat in rodents can increase neuroinflammation and promote the expression of inflammatory markers in the hippocampus, which are, in turn, associated with spatial working memory impairment. The effects of the diet on behaviour were not persistent when the diet was reversed to a standard diet in adulthood [52]. A poor-quality diet high in fat and sugar is associated with higher rates of inflammatory cytokines [32]. Examining the role of inflammation in the association between obesity and depression demonstrated a correlation between neuroinflammation induced by body weight in adolescence and low mood [53]. 

MFGM supplementation influences the gut microbiome and mood regulation [54,55,56]. The gut microbiota mediate the digestion of food and dietary supplements and influence the production of neurotransmitters [57]. Evidence suggests that MFGM supplementation influences the synthesis and action of neurotransmitters implicated in different psychological states through their interactions with the gut microbiota [58]. Lactic acid bacteria fed MFGM showed a significant increase in the production of serotonin [59], suggesting that MFGM supplementation in animals and humans may influence existing lactic acid-producing bacteria in the gut microbiome to produce serotonin. Furthermore, individual components of the MFGM can influence the synthesis of neurotransmitters implicated in mood and cognition. For example, phosphatidylserine affects serotonin and dopamine production and activity via gut microbiota modifications [60,61], and phosphatidylcholine is required to synthesise the neurotransmitter acetylcholine [62]. The abundance of bifidobacteria and lactic acid bacteria increases with MFGM supplementation [2]. In a rodent model of stress, supplementation with the MFGM product Lipid 70 [63] and *L. rhamnosus* HN001 synergistically altered GABA subunit expression in the limbic system, specifically the amygdala and hippocampus. Lipid 70 supplementation was independently associated with a shift in the microbiota of the large intestine and short-chain fatty acid levels [64]. However, supplementation with either product in isolation or conjunction was not associated with changes in anxiety, depression, or stress symptoms. Although the mechanisms underlying potentially synergistic effects of MFGM and probiotics are not well elucidated, the MFGM may assist the psychobiotic effects of probiotic supplements by protecting the passage of the probiotic through the gastrointestinal tract and interaction with the mucosal cells in the gut [54,65,66,67].

## 3. Mental Health in Infancy, Childhood, and Adolescence

Milk fat globule membranes have the potential to support optimal behavioural and emotional development and to ameliorate the symptoms of neurodevelopmental and psychiatric conditions due to their rich composition of nutrients essential for brain health. Synaptogenesis and myelination require lipids to support optimal neuronal development and connectivity [68]. Rapid brain growth and maturation in infancy and early childhood and accelerated maturation of the pre-frontal cortex in adolescence are mirrored by concurrent cognitive skill acquisition and emotional development. Mental health disorders predominantly emerge between childhood and early adulthood [17], highlighting the importance of optimising early brain health for good mental health throughout the human lifespan.

### 3.1. Pre-Clinical Models of Early MFGM Supplementation for Emotion Regulation

Pre-clinical models link early MFGM supplementation with alterations in the brain lipidome, indicating that lipids provided in nutritional supplements can alter brain polar lipids [69,70]. A rat model of postnatal supplementation with a whey-based MFGM formulation through to young adulthood modelled the potential effects of long-term supplementation on adult outcomes. Early-life MFGM supplementation improved spatial memory into adulthood and altered expression in emotional circuit pathways, reducing anxiety in juvenile rats [71]. In young piglets, a combination of MFGM, lactoferrin and dietary prebiotics supplementation altered the micro-structural maturation of the brain in the internal capsule and cortical regions [72]. Testing three doses of MFGM supplementation in neonatal piglets, MFGM was associated with improved spatial learning on the T maze test with the possible mechanism of improved white matter connections in the brain, as measured by fractional anisotropy in the hippocampi [66]. 

MFGM supplementation during sensitive periods of development in early life may improve the function of the stress response system later in life. Visceral pain sensitivity induced by stress exposure was reduced with MFGM supplementation in rats exposed to early stress from maternal separation [73]. Early MFGM supplementation was associated with an altered endocrine response to acute stress in chronically stressed mice [15]. In non-stressed mice, supplementation reduced levels of innate anxiety behaviour. By contrast, supplementation in young adulthood reversed anxiety signs in mice exposed to chronic stress. The results suggest that different supplementation periods may exert differential effects on emotion and behaviour regulation in response to stress. Not all studies show an associated change in behavioural or cognitive outcomes, suggesting a complex array of mechanisms underpinning mental health in early life and beyond [70,74]. Longer-term effects on psychiatric outcomes may be subtle and more difficult to detect [75]. 

### 3.2. The Use of the MFGM to Support Behavioural and Emotional Development in Early Life

The development of typical behavioural and emotional skills is a key feature of early life associated with rapid central nervous system development that underpins mood stability later in life. Breastmilk is the gold standard of infant nutrition and is rich in nutrients and MFGMs [76]. Fortification of infant formula aims to bring formula composition closer to that of breastmilk [77,78]. Term infants fed a phospholipid-enriched formula had more advanced neuronal myelination than infants fed standard formula [79]. Early cognitive, behavioural, and emotional development appears to be advanced in children receiving enriched formula compared to standard formula for outcomes measured concurrently with supplementation. Infants supplemented with MFGM-enriched formula compared with those on standard formula had higher Bayley Scales of Infant Development composite scores at 12 months of age and significantly higher social emotional scores and general adaptive scores on a parent-report measure of self-care and independence, and significantly higher short-term memory [80]. In another study, infants fed formula supplemented with MFGM-enriched or standard formula were compared with breastfed infants at 18 months and 2.5 years using the Child Behaviour Checklist. Infants receiving the enriched formula had fewer affective problems and externalising behaviours than those fed a standard formula [81]. Infants given formula enriched with bovine lactoferrin and MFGMs had higher language and motor index scores on the Bayley Scales at 12 months [61]. A follow-up assessment of the cohort at 5.5 years found that children who had received the enriched formula continued to have higher cognitive development scores. However, there was no significant difference in the Child Behaviour Checklist between the groups, suggesting that despite persistent cognitive benefits, behavioural development may be more complex, including how it is influenced by nutrition in the first year [82,83]. Similarly, infants supplemented with an MFGM-enriched formula from 2 months had significantly higher neurodevelopmental scores on the Bayley Scales of Infant and Toddler Development at 12 months [84].

A later follow-up of the cohort at school age found no significant difference between the groups on any cognitive or behavioural measures. The authors speculate that this could have been due to other environmental influences on development between 12 months and 6.5 years or that the study was underpowered to detect differences in outcome [85]. In an older preschool sample (mean age 4.4 years), children were randomised to receive a chocolate milk drink fortified with MFGMs or a standard chocolate drink daily for four months. Parents and teachers reported internalising and externalising problems using the validated Achenbach questionnaires. Supplemented children had fewer externalising and internalising problems and lower overall problem scores [86]. These results, as well as those from studies showing enhanced neurodevelopment measured temporally close to the supplementation period, suggest that MFGM supplementation may improve behavioural and emotional development during the intervention period. Evidence to support persistent positive effects on behavioural development when supplementation has ceased needs to be clarified.

### 3.3. Neurodevelopmental Disorders

Attention Deficit Hyperactivity Disorder (ADHD) is a neurodevelopmental disorder arising in childhood characterised by difficulties with attentional control, including problems sustaining, dividing, or switching focus, impulsivity, and hyperactivity [87]. These symptoms can interfere with a child’s learning progress in the educational domain, and there is considerable interest in exploring nutritional interventions for ADHD. Children with ADHD have reduced PS concentrations in key brain regions involved in attention and executive control, including the basal ganglia and pre-frontal cortex, providing a rationale for phospholipid supplementation in the treatment of ADHD [88,89]. Early rodent model studies indicate that phosphatidylserine specifically may modulate neurotransmitter release [90,91]. In an intervention trial, children aged between 4 and 14 years with diagnosed ADHD and no history of medication for the disorder were randomly allocated to receive a soy-derived phosphatidylserine supplement or placebo for two months. Supplemented young people had improved auditory memory scores and a reduction in ADHD symptoms of both the inattentive and hyperactive/impulsive types [92]. Manor et al. in 2013 used a PS supplement with omega-3 for 15 weeks in a double-masked controlled study followed by a further 15-week open trial; their results found improvements in ADHD symptoms, specifically in parent-reported impulsivity and behaviour regulation [93]. While these results suggest that PS may be beneficial for the externalising aspects of ADHD, including restlessness and regulation of outward behaviour, in a more recent review on the topic, four studies investigating PS supplementation for symptoms of ADHD met the inclusion criteria. PS was associated with minor improvements in symptoms. Meta-analyses of three studies found a small but statistically significant reduction in symptoms of inattention associated with PS supplementation. Total ADHD symptom scores and hyperactivity score differences did not reach statistical significance [94]. The authors rated the overall quality of current trials of phosphatidylserine in ADHD as low due to methodological issues [90]. There is a lack of studies investigating the potential for the MFGM as a whole compound to treat the symptoms of ADHD.

Autism Spectrum Disorder (ASD) is a neurodevelopmental disorder with symptoms occurring along a spectrum characterised by social communication problems and stereotypical or repetitive behaviours. Gastrointestinal symptoms and altered gut microbiota are further features consistently associated with ASD [95]. Plasma concentrations of different phospholipids (PLs) were demonstrably lower in a sample of people with ASD from Saudi Arabia, leading the authors to suggest that early PL supplementation may increase plasma and brain PL concentrations, promoting an anti-neuroinflammatory response [96]. Antiphospholipid antibodies were elevated in a sample of young children with ASD when compared with typically developing controls and those with non-autistic developmental delays, suggesting that PLs may play a role in the pathogenesis of ASD symptoms. Further, increases in PL antibodies were associated with more prominent ASD symptoms [97]. To date, the role of PLs and their related metabolites in ASD has focused primarily on identifying biomarkers that could support the diagnosis and assessment of ASD symptoms [98], and randomised trials of the MFGM in ASD are not present in the literature. Recent examination of the developing rat brain shows a dynamic lipid profile altered by PL supplementation, resulting in observable changes in the lipid profile of different brain regions. This pre-clinical lipidomic evidence provides a rationale for lipid supplementation to influence neurodevelopment [99].

## 4. MFGM and Adult Mental Health

### 4.1. Pre-Clinical Models of Mood Disorders

MFGM supplementation alters the phospholipidomic profiles in the brain of rodent models of depression [100,101]. Rats supplemented with phosphatidylserine purified from bovine brain tissue swam longer in the Forced Swim Test, demonstrating an antidepressive effect [102]. Both anxiety and stress are emotional responses to situational events and exhibit intertwined behavioural and neural underpinnings [103]. Stress induces changes to the gut microbiome, making the gut microbiota and stress direct targets for intervention [43]. Enhanced gene expression in the brain of rats supplemented with a probiotic and MFGM demonstrates the synergistic action of the two on neural pathways associated with anxiety, fear and depression [64]. In other animal models, phospholipids supplemented via a buttermilk concentrate exert protective effects against chronic stress and anxiety-like behaviours [15]. Specifically, changes in the levels of the phospholipids phosphatidylcholine (PC) and phosphatidylethanolamine (PE) have been observed in the brains of mice [100] and rats [101] after exposure to unpredictable stress conditions. Conversely, phospholipid supplementation of mice with depleted phospholipid concentrations in the brain restored the deficits in phospholipid composition, which was further associated with improved cognitive learning [104].

### 4.2. Evidence from Clinical Trials in Human Populations

In human participants, the response to situational stress improves in those supplemented with MFGMs [105,106]. Healthy adults aged 25–65 years who received daily MFGM supplementation had significantly lower stress scores and a borderline significant reduction in anxiety symptoms compared to participants receiving the placebo [107]. Men supplemented with a milk-derived phospholipid concentrate show stress reduction, specifically attenuation of stress-induced memory impairments [108], improved post-stress reaction time performance on an attention-switching task [109] and blunted psychological stress response when exposed to a high-stress load [106]. Phosphatidylserine alone improves mood and feelings of stress, as well as heart rate responses to acute stressors in young adults with neuroticism [110]. 

## 5. MFGM and Mental Health in Older Adults

Cognitive decline can occur as the result of general age-related changes or in the presence of a dementia diagnosis. Researchers typically define cognitive impairment as a memory impairment beyond that expected for age and education [111]. Recent definitions suggest cognitive impairment as ‘evidence of impairment on three consecutive neurological or neuropsychological assessments’ [112]. Cognition can be differentiated from mental disorders in older adults. Mental illness refers to the presence of psychiatric disorders such as depression and anxiety [113]. Symptoms can include disengagement, lingering low mood, and a heightened sense of fear [114]. Neurological processes influence cognition and mental health, where interactions between specific brain regions mediate emotional influences on cognitive functions, such as decision-making and the regulation of emotion [115]. Therefore, dementia can impact mood and cognition [116,117]. In older adulthood, age-related cognitive decline is typical, partly due to the depletion of polyunsaturated fatty acids (PUFAs) and phospholipids in the CNS [118]. Given their demonstrated benefits on cognitive development in infants, it is unsurprising that there is increasing interest in using MFGM supplementation for brain health in older adults, specifically in age-related cognitive decline and in dementia, where mood disturbances are common [119,120].

### 5.1. Pre-Clinical Studies in Aged Rodent Models

Aged rats supplemented with MFGMs had improved vascular density, dopamine production and improved in measures of neuroplasticity and memory with scores closer to those of younger rats. These results suggest that supplementation early in ageing may prevent memory decline through improved vascular and neuronal functioning [121]. In a series of studies using MFGMs from a concentrated buttermilk extract in aged rodents, MFGM supplementation modulated gene expression in the hippocampus, specifically in genes involved in pathways related to Alzheimer’s Disease [122]. MFGM supplementation increased levels of EPA and DHA fatty acids in the synaptosomes extracted from the hippocampus [123], and MFGMs improved synaptic signalling in the brain [124]. These findings combined suggest that MFGM supplementation could restore the cerebral connections in the brain to prevent or slow age-related cognitive decline. Using a dose of 70 mg MFGM in rats, which is consistent with a ‘typical’ adult dietary dose, a positive reversal of some of the parameters of age-associated behaviours of emotional memory was observed in rats [125]. More recently, supplementation with an MFGM-rich whey protein powder improved the cognitive ability of a mouse model of Alzheimer’s disease via modulation of neuroinflammatory pathways in the brain [126].

### 5.2. Clinical Trials in Older Adults

In adults aged 65 years and older with a mild age-related cognitive impairment, supplementation of MFGMs as a fortified milk drink improved short-term memory, specifically episodic memory or the ability to recall recent events in female participants [127]. Loneliness, anxiety, and late-life depression are acutely associated with a perceived decline in cognitive function [128,129]. If MFGM intake improves cognitive ability, this may have positive sequelae for mood. MFGM supplementation may directly improve mental health through similar mechanisms to those observed in studies of adults with symptoms of anxiety, depression, or stress. In older women with MDD, supplementation with phosphatidylserine at a daily dose between 300 mg and 600 mg significantly improved symptoms of depression assessed using the Hamilton Rating Scale [130,131,132]. Phosphatidylserine supplementation of 300 mg daily in non-depressed elderly participants resulted in fewer depressive symptoms and ‘winter blues’ measured using the List of Depressive Symptoms and Hamilton Rating Scale [132,133]. Furthermore, when combined with EPA and DHA fatty acids, phosphatidylserine supplementation significantly lowered symptoms of depression in older people with MDD [134].

## 6. Conclusions

Milk fat globule membranes have the potential to alter psychological outcomes modulated by the gut–brain axis and pathways of neurotransmitter synthesis and immune-mediated effects on chronic inflammation. Understanding the bioactive properties of the MFGM and their actions in the gut and CNS is crucial in realising the role of MFGMs in preventing or treating mental health disorders in clinical populations. Clinical trials in healthy volunteers should examine the potential for enhancing psychological wellbeing more generally.

The persistence of the observed benefit for behavioural development in preschool children supplemented with MFGMs requires longitudinal research. Adolescence represents a sensitive period for brain maturation and myelination of the pre-frontal regions and mental health disorders commonly emerge in adolescence. MFGMs represent an unexplored intervention for supporting adolescent mental health and development. Shared neural underpinnings of cognitive and mental function provide a rationale for future clinical trials to investigate the potential of MFGMs for improved psychological outcomes in adults and those of older age.

## Data Availability

No new data were created or analyzed in this study. Data sharing is not applicable to this article.
